# Eclipsed mitral regurgitation and the role of multimodality imaging: a case report

**DOI:** 10.1093/ehjcr/ytaf075

**Published:** 2025-02-25

**Authors:** Stavros Kounas, Nektarios Pilatis, Eirini Solomou, Markos Koukos, Maria Aroni

**Affiliations:** 5th Cardiology Department-Heart Valve Center, Henry Dunant Hospital, Mesogion 107, Athens 11 527, Greece; 5th Cardiology Department-Heart Valve Center, Henry Dunant Hospital, Mesogion 107, Athens 11 527, Greece; 5th Cardiology Department-Heart Valve Center, Henry Dunant Hospital, Mesogion 107, Athens 11 527, Greece; 5th Cardiology Department-Heart Valve Center, Henry Dunant Hospital, Mesogion 107, Athens 11 527, Greece; 4th Cardiothoracic Surgery Department-Heart Valve Center, Henry Dunant Hospital, Mesogion 107, Athens 11 527, Greece

**Keywords:** Transient mitral valve regurgitation, Acute pulmonary oedema, Eclipsed MR, Coronary vasospasm, Multimodality imaging, Exercise imaging tests

## Abstract

**Background:**

Eclipsed mitral regurgitation (MR) is a rare, reversible condition that leads to transient severe MR and acute heart failure in patients with preserved left ventricular (LV) ejection fraction. Its diagnosis is challenging due to its intermittent presentation, necessitating advanced imaging techniques to reveal the underlying pathology.

**Case summary:**

We present a case of a 74-year-old female with recurrent severe MR associated with a non-obstructive lesion in the proximal left anterior descending (LAD) artery. Multimodality imaging played a pivotal role in diagnosing this condition, as conventional vasodilator stress tests failed to uncover the ischaemic mechanism. Exercise stress echocardiography and myocardial perfusion scintigraphy successfully demonstrated a small ischaemic region affecting the mitral valve apparatus, which triggered severe MR during physical exertion. This dynamic ischaemia, undetected by routine tests, was essential in diagnosing the pathophysiology behind the patient’s recurrent MR. Following percutaneous coronary intervention (PCI) to the LAD, her symptoms resolved, confirming the ischaemic origin of the MR.

**Discussion:**

This case underscores the critical role of multimodality imaging in revealing the pathophysiology of recurrent MR. Advanced imaging techniques, particularly under physiologic stress, are crucial for diagnosing dynamic ischaemia and its impact on valvular function. By identifying the ischaemic cause of MR, individualized treatment strategies, such as PCI, can be implemented, avoiding unnecessary valve surgery and improving patient outcomes.

**Learning points**

This case highlights the following:

1. Eclipsed mitral regurgitation (MR) can be an often overlooked cause of recurrent pulmonary oedema in patients with preserved ejection fraction.
2. Even small regions of left ventricular ischaemia, when involving the mitral valve apparatus, can lead to severe heart failure symptoms, though this is rare.
3. Transient severe functional MR may be partly due to coronary vasospasm, meaning that non-physiological or vasodilatory stress tests may fail to detect the underlying pathology.
4. Elucidating the causative mechanism in mitral valve regurgitation is crucial in order to offer the appropriate personalized therapy.

## Introduction

Eclipsed mitral regurgitation (eclipsed MR) is a rare condition characterized by completely reversible severe functional MR, leading to acute heart failure in patients with a normal left ventricular (LV) ejection fraction (EF). The term was first introduced by Avierinos *et al*. in 2008, who reported three patients with transient, massive functional MR in the absence of pre-existing LV dysfunction or significant coronary artery disease (CAD). The reversible nature of the condition makes it difficult to recognize, and its underlying causes are multifactorial. This is evidenced by the small number of case reports over the years, with patients being diagnosed and managed in different ways—from medical management to surgical valve replacement and transcatheter repair—depending on the underlying pathophysiology.

We present a compelling case of dynamic MR caused by less than 10% ischaemia in a region of the left ventricle due to a non-obstructive lesion in the left anterior descending (LAD) artery, affecting the mitral valve (MV) apparatus. While functional imaging with vasodilatory stress tests failed to reveal the underlying pathology, the use of multimodality imaging, specifically exercise-based tests, successfully identified the subtle ischaemia responsible for the mechanical dysfunction of the MV. This case underscores the importance of multimodality imaging when conventional methods fail to uncover the cause of severe intermittent regurgitation.

## Summary figure

**Figure:**
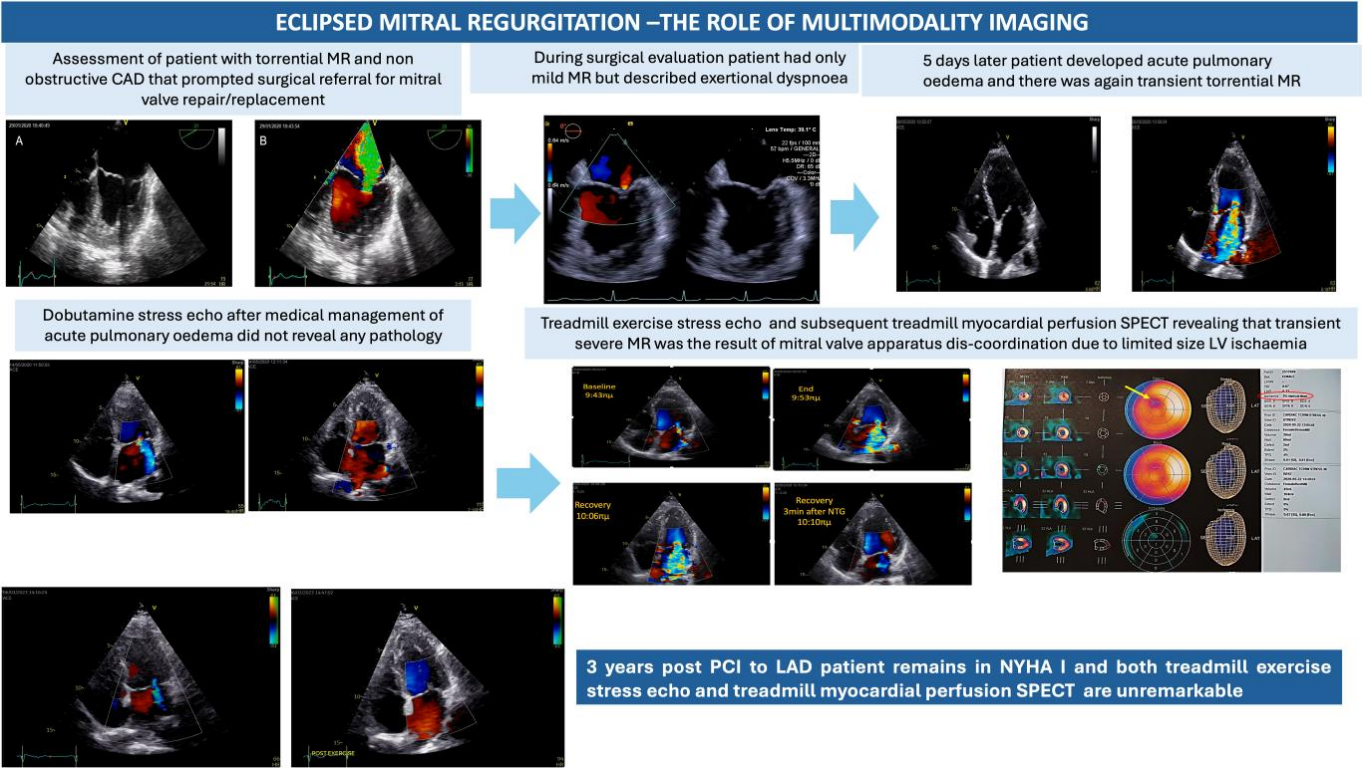


## Case presentation

We revisit the recent medical history of a 74-year-old female patient with a background of primary sclerosing cholangitis, paroxysmal atrial fibrillation, and a history of nephrolithiasis. One year before presenting at our centre, she was admitted to another hospital with a urinary tract infection complicated by acute respiratory failure of unclear aetiology, necessitating intubation. She had a short stay in the intensive care unit and was extubated within 24 h. After discharge, she experienced repeated episodes of transient dyspnoea and chest discomfort, with a concomitant drop in oxygen saturation (SO_2_ < 90%) on pulse oximetry.

**Figure 1:**
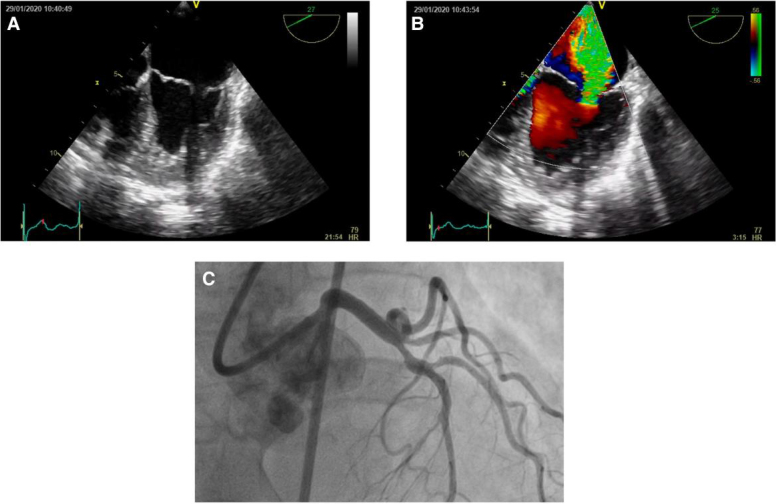
Initial transoesophageal echo and diagnostic coronary angiogram revealing severe mitral valve regurgitation. (*A*) 0–30 degrees four-chamber view in end-systole revealing coaptation gap of mitral valve leaflets. (*B*) Same image with colour revealing severe mitral regurgitation. (*C*) Diagnostic coronary angiogram right anterior oblique cranial view demonstrating 60–70% disease on proximal left anterior descending artery and 70% on ramus intermedius branch (small vessel).

A few months later, she was re-admitted to the same hospital due to chest pain and acute pulmonary oedema. Diagnostic workup revealed an unremarkable 12-lead electrocardiogram (ECG) and negative biomarkers for myocardial injury (hs-troponin). However, a transthoracic echocardiogram (TTE) revealed severe functional MR, later confirmed by transoesophageal echocardiogram (TOE) (*Figure 1A* and *B*). During that time, the patient was in normal sinus rhythm and her blood pressure remained within normal range. A coronary angiogram showed intermediate proximal LAD stenosis (*Figure 1C*), which was deemed non-obstructive after fractional flow reserve (FFR) assessment (FFR = 0.86). Consequently, the patient was referred for surgical treatment of MV disease, with the option of a left internal mammary artery graft to the LAD. The patient had received recommendations for MV replacement from two different cardiothoracic centres before seeking a third consultation at our centre.

**Figure 2:**
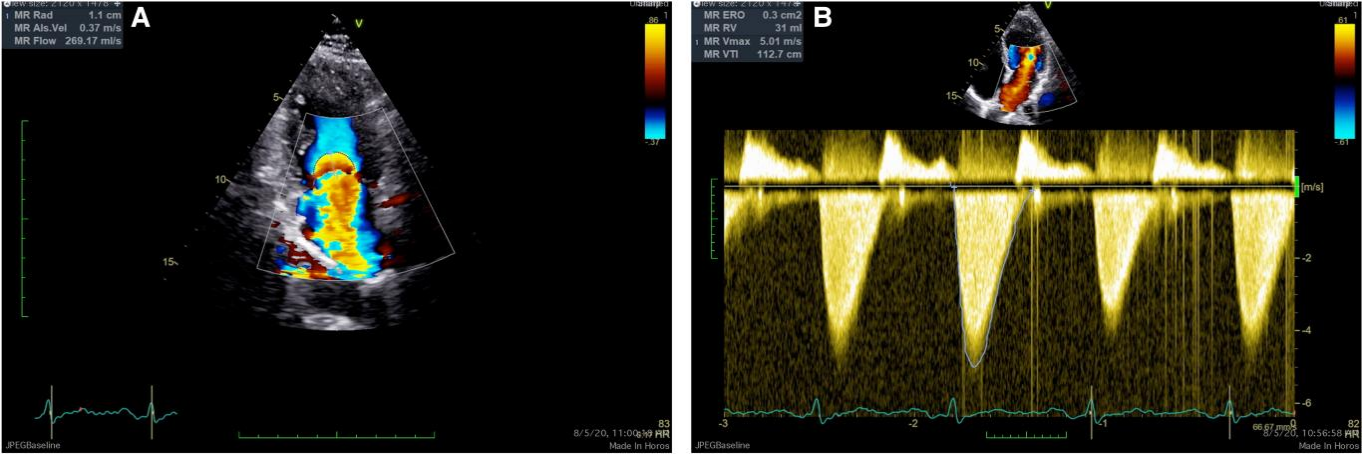
Recurrence of severe mitral regurgitation with validated quantification in transthoracic echocardiogram. (*A*) Pisa mitral regurgitation 1.1 cm indicating severe regurgitation. (*B*) Effective regurgitant orifice area and regurgitant volume measured with continuity method. Note that the triangular shaped envelope of mitral regurgitation indicates acute severe mitral regurgitation and that the effective regurgitant orifice area and regurgitant volume could be underestimated since the continuity equation assumes a more uniform flow.

Pre-operative evaluation was performed at our Heart Valve Center, including a new TTE/TOE, per our practice protocol, to assess the valve anatomy and the mechanism of regurgitation. To our surprise, the assessment revealed only mild MV regurgitation (see Supplementary material online, *Videos S1–S5*). As a result, an exercise echocardiogram was ordered to evaluate a possible dynamic component. However, 5 days later, the patient presented with symptoms and signs of evolving pulmonary oedema during her scheduled appointment. The new TTE at that time showed massive MV regurgitation with triangular regurgitant jet in continuous wave Doppler (*Figure 2*), with systolic mitral leaflet restriction, causing a lack of coaptation (see Supplementary material online, *Videos S6* and *S7*), as well as severely elevated systolic pulmonary artery pressure, demonstrated in comparison with her initial assessment (see Supplementary material online, *Figure S1*). The patient’s chest X-ray is shown in Supplementary material online, *Figure S2*. It is worth noting that no fluctuations in blood pressure were observed between assessments and that she was always in sinus rhythm and the exact vital signs are documented in the figure legends of each assessment. The patient was admitted for heart failure treatment with intravenous (IV) diuretics.

**Figure 3:**
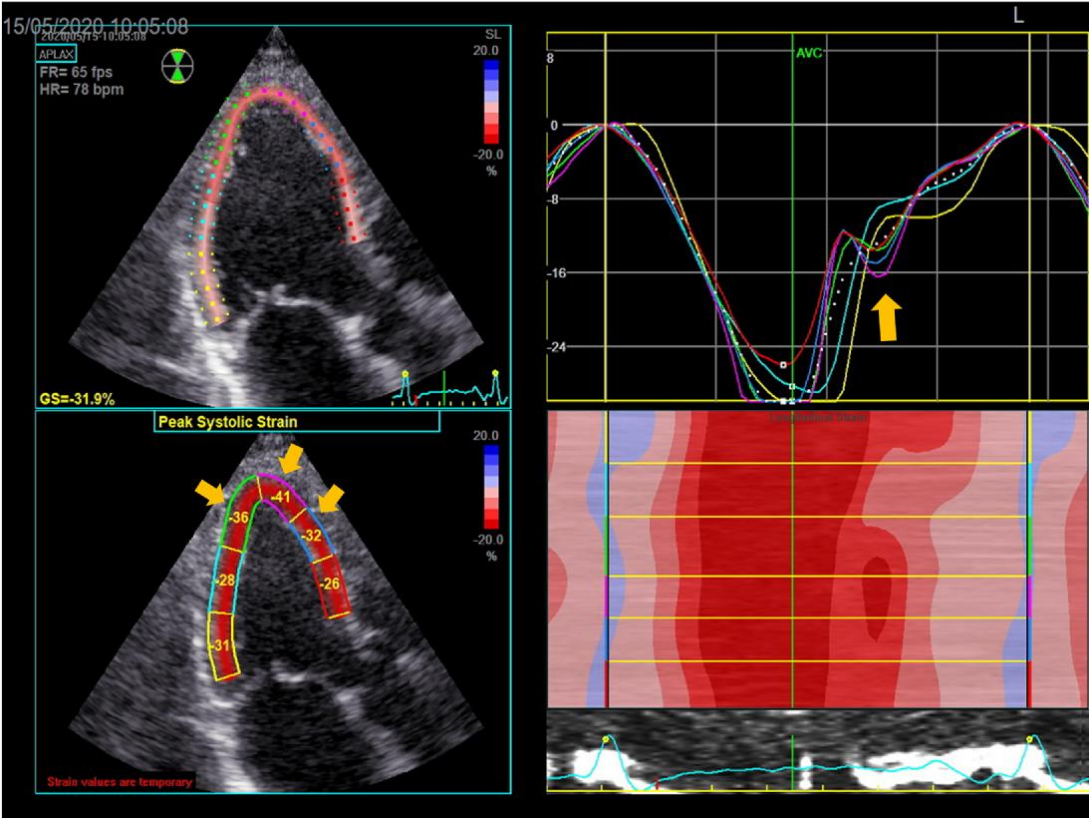
Left ventricular longitudinal strain at baseline and at peak exercise. Mid**-**apical antero-septal post-systolic shortening at peak stress compared to baseline is demonstrated with arrows. Strain curves normalized after the administration of sublingual nitrates.

**Figure 4:**
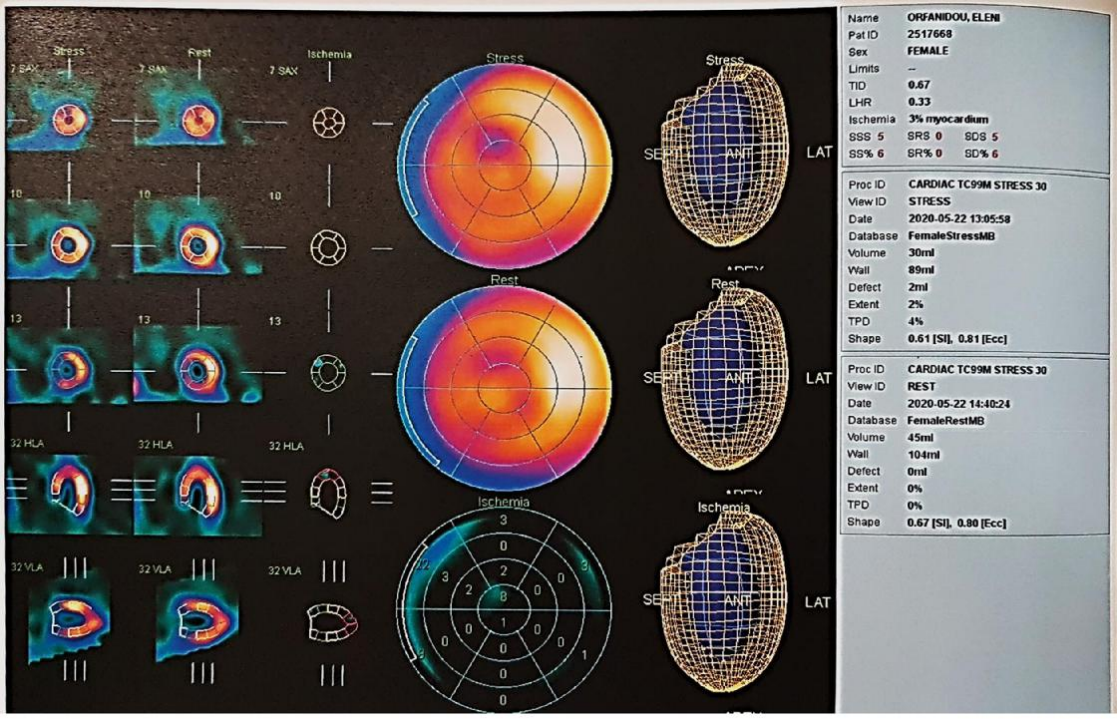
Single-photon emission computed tomography with exercise on treadmill. Three per cent area of ischaemia on the apical anterior wall.

During admission, after stabilizing the patient, we assessed the coronary flow reserve of the LAD using IV adenosine, which revealed normal findings (see Supplementary material online, *Figure S3*). We proceeded with a dobutamine stress echocardiogram to check for myocardial ischaemia, which returned negative results, showing no regional wall motion abnormalities at peak stress (>85% of the age-predicted heart rate) (See Supplementary material online, *Videos S8–S13*). We then followed through with the initial plan for treadmill exercise echocardiography. Pre-exercise TTE showed mild MR (*Video 1*). The patient managed 4 min of exercise on the treadmill (modified Bruce protocol) before stopping due to dyspnoea and angina. Electrocardiogram recordings at the time were unremarkable (see Supplementary material online, *Figure S4*). Subsequent TTE revealed torrential MR with minimal improvement after 5 min of recovery (*Video 2*; see Supplementary material online, *Video S14*). Despite a blood pressure reading of 110/70 mmHg, sublingual nitroglycerine was administered, leading to an immediate resolution of MR and her symptoms (*Video 3*). Left ventricular speckle-tracking longitudinal strain analysis showed normal rest and peak EF and global longitudinal strain values, but regional strain curve analysis revealed reversible post-systolic shortening of the mid-apical antero-septal segments, indicating subtle ischaemia over the distal LAD region (*Figure 3*). To support these findings, a myocardial perfusion single-photon emission computed tomography (SPECT) using a modified Bruce treadmill protocol was performed. The test was again prematurely terminated due to symptoms, but scintigraphy images revealed a 3% area of LV ischaemia in the apical anterior wall (*Figure 4*).

**Figure 5:**
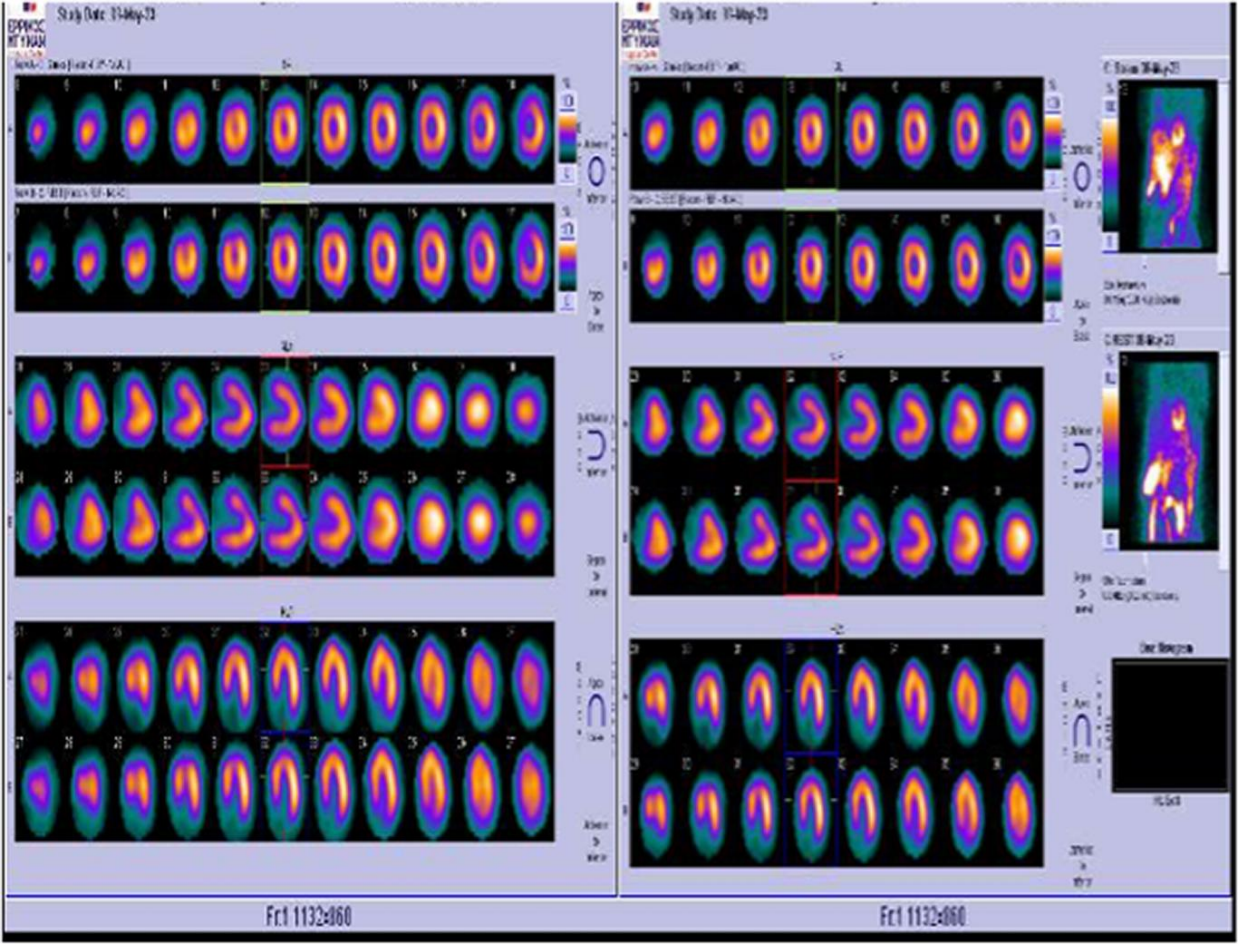
Single-photon emission computed tomography with exercise on treadmill post-revascularization.

After a short period of treatment with vasodilatory agents (nitrates and diltiazem), during which symptoms remained unchanged, the patient underwent percutaneous coronary intervention (PCI) to the proximal LAD (see Supplementary material online, *Videos S15* and *S16*). Since then, she has remained asymptomatic, in New York Heart Association (NYHA) Class I. Three years after PCI, follow-up exercise echocardiography and exercise SPECT testing showed normal findings (Supplementary material, *Videos S17* and *S18*; Supplementary material online, *Figure S5*; *Figure 5*).

## Discussion

Eclipsed MR is a rare and often under-recognized clinical phenomenon that can lead to recurrent episodes of acute pulmonary oedema in patients with preserved LV EF.^1,2^

This case exemplifies how small, localized areas of myocardial ischaemia can severely disrupt the coordination of the MV apparatus, leading to intermittent MR.^2,3^ The ischaemic insult in such patients may not be widespread, making it difficult to detect using traditional imaging techniques like vasodilatory stress testing or dobutamine echocardiography. As was seen in this case, these methods often miss the nuanced pathophysiology due to their non-physiologic nature. Our findings indicate that transient severe MR can arise due to mechanical discoordination of the MV apparatus caused by subtle ischaemia of the mid-apical anteroseptal segments, preventing proper leaflet coaptation. This was also supported by the reversible post-systolic shortening observed during strain imaging.

The role of coronary vasospasm in exacerbating ischaemia is another critical point highlighted by this case. This phenomenon could explain why certain non-invasive tests failed to replicate the pathophysiological state that produced the severe MR. Vasospasm may not always be induced during routine testing, especially when using pharmacological agents that mimic, but do not fully reproduce, physiological stress.^4^ This insight suggests that vasospasm should be more routinely considered in cases where patients present with symptoms that do not correlate with typical ischaemic findings.

In the context of eclipsed MR, distinguishing between various causes of transient MR is crucial for accurate diagnosis and management. Eclipsed MR is characterized by severe, yet reversible, MR that occurs under specific physiological stress, such as exercise, and resolves at rest, making it challenging to diagnose with conventional methods. Differential diagnosis should consider several key causes of transient MR, including ischaemic MR, functional MR due to LV dysfunction, pappilary muscle dysfunction, and transient increases in left atrial pressure, which may all present with episodic MR. This differentiation is essential, as it directs treatment strategies—whether addressing ischaemic mechanisms, improving haemodynamics, or intervening surgically—towards the underlying cause of transient MR, thereby preventing mismanagement or unnecessary valve interventions.

Exercise-based multimodality imaging proved to be the key diagnostic modality that successfully uncovered the ischaemia responsible for the patient’s recurrent pulmonary oedema and MR.^4^ The use of treadmill exercise stress testing, coupled with echocardiography and myocardial perfusion scintigraphy, allowed us to simulate the physiological conditions under which the patient’s symptoms manifested. These dynamic tests are able to highlight transient ischaemia, which might be missed during resting or pharmacologically induced stress tests.

Moreover, this case underscores the importance of personalized therapy based on individualized diagnostic findings. In the era of precision medicine, identifying the specific mechanisms behind a patient’s disease allows for more targeted interventions.^5^ The use of PCI to address the proximal LAD lesion resulted in complete symptom resolution, which further emphasizes the need for tailored treatment strategies in such complex cases. Not all patients with functional MR require valve replacement or repair; rather, identifying and treating the underlying aetiology can lead to excellent clinical outcomes, as seen in this patient who remained asymptomatic for 3 years post-PCI.^4^

This case raises the possibility that eclipsed MR may be more prevalent than currently recognized. The intermittent nature of the condition and the reliance on non-physiological testing likely contribute to its underdiagnosis.^3,5^ Further research and awareness are needed to refine diagnostic criteria, develop more sensitive testing protocols, and explore whether specific subpopulations may be more susceptible to this condition. The potential role of coronary vasospasm in other forms of dynamic valvular dysfunction also warrants further investigation.

## Limitations

This case report has a few limitations. Although coronary vasospasm was not confirmed through a reactivity test or the presence of ST-elevation on the ECG—both of which are typically associated with vasospasm-induced ischaemia—the transient nature of the ischaemia and MR, the discrepancy between exercise and pharmacological stress testing, the absence of significant coronary obstruction, and the rapid response to nitrates strongly suggest that coronary vasospasm was the underlying cause of the acute event.

Additionally, cardiac magnetic resonance imaging (CMRI) was not performed due to the clinical urgency and evolving symptoms, which necessitated prompt diagnosis and treatment using other multimodality imaging techniques. However, CMRI could have offered further insights, particularly in detecting microvascular dysfunction, potentially contributing to a more comprehensive understanding of the ischaemic events.

## Conclusion

In conclusion, eclipsed MR represents an important and often overlooked contributor to acute heart failure in patients with preserved EF. This case highlights the utility of exercise-based multimodality imaging in uncovering the underlying ischaemia and emphasizes the importance of individualized treatment approaches. Future studies should aim to refine diagnostic strategies to better identify and manage this elusive but clinically significant condition.

## Supplementary Material

ytaf075_Supplementary_Data

## Data Availability

The data underlying this article are available in the main manuscript or the supplemental material. Due to ethical and privacy concerns, some supporting data cannot be made publicly available but are available from the corresponding authors upon reasonable request.

## Lead author biography

Dr Stavros Kounas is a cardiologist, Director of the Echocardiography at the Henry Dunant Hospital Center in Athens, Greece. He is specialized in cardiovascular imaging and works as a member of the Hospital’s Heart Team managing patients with complex valvular disease. He holds EACVI accreditation in TOE and TTE since 2008. In the past, he has been occupied as a cardiology registrar at the Echocardiography Department of The Heart Hospital, UCLH, London, UK, and as a research fellow in the Cardiomyopathy Clinic under Professors W. McKenna and P. Elliott.

## Notes

**Consent:** Written informed consent for publication of anonymized data was obtained from patient.

**Funding:** None declared.
