# Supplementary figures and images for: Eclipsed mitral regurgitation and the role of multimodality imaging: a case report
Source: Eur Heart J Case Rep. 2025 Feb 25;9(3):ytaf075. doi: 10.1093/ehjcr/ytaf075 (PMC11997780; doi:10.1093/ehjcr/ytaf075)

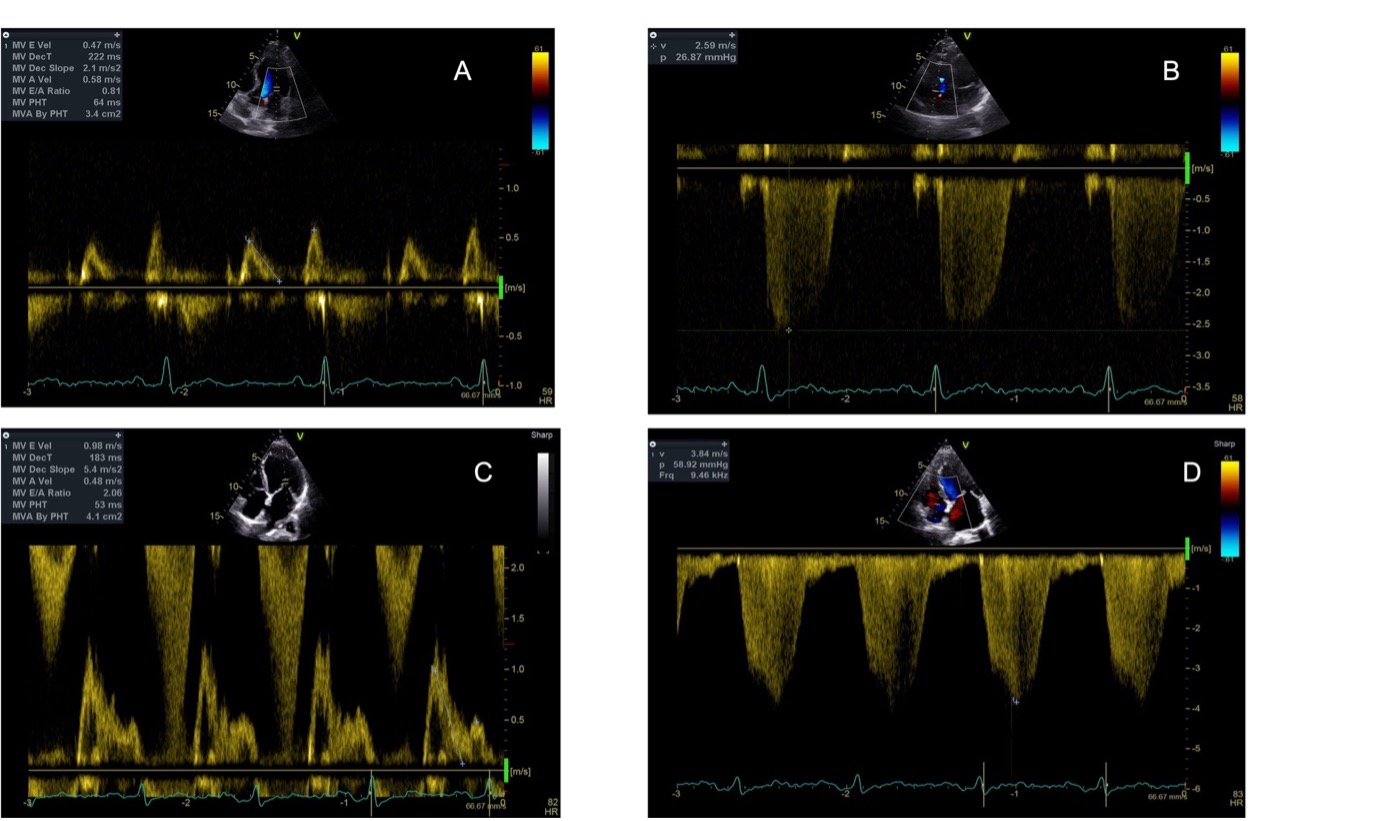

Supplement: ytaf075_Supplementary_Data [file ytaf075_Supplementary_Data.zip › sup figure 1.jpg]

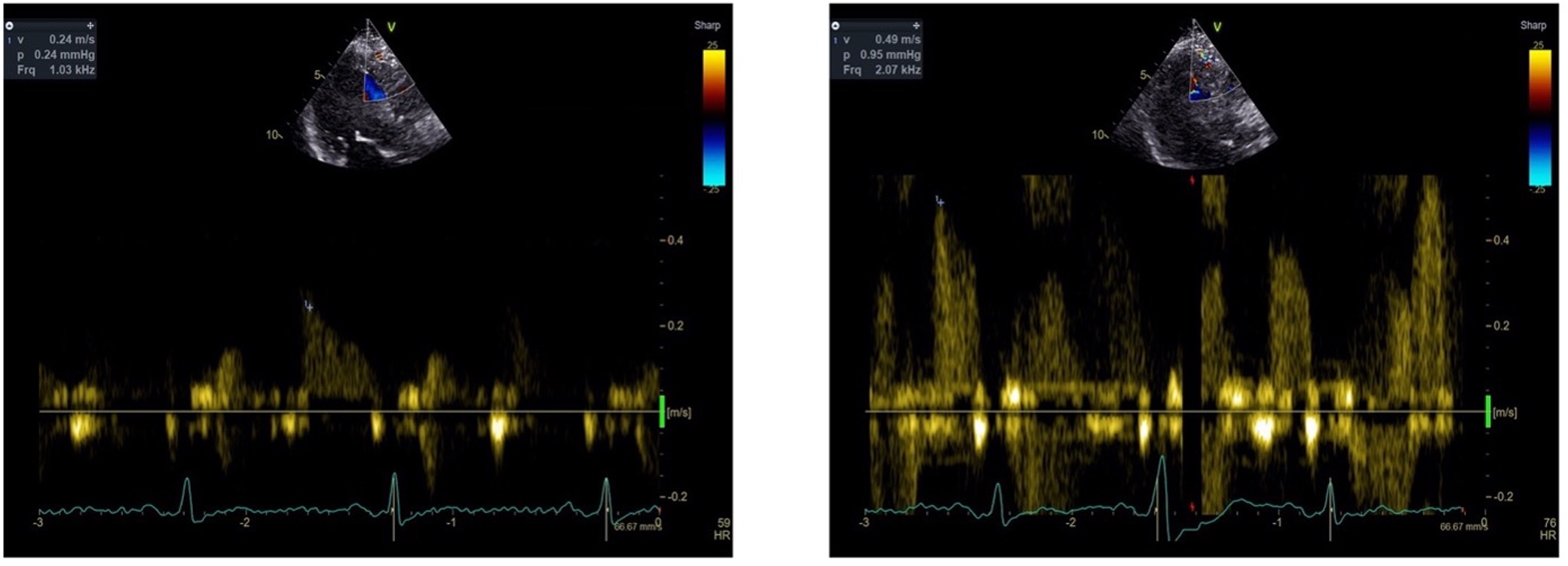

Supplement: ytaf075_Supplementary_Data [file ytaf075_Supplementary_Data.zip › sup figure 3.jpg]

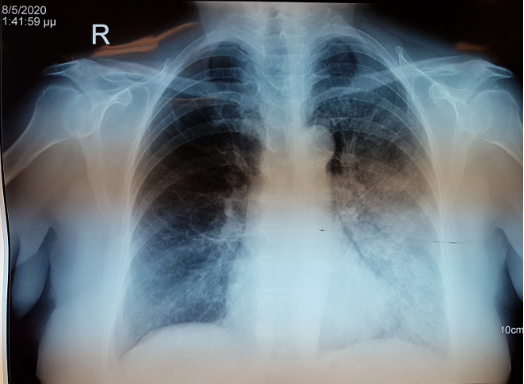

Supplement: ytaf075_Supplementary_Data [file ytaf075_Supplementary_Data.zip › Sup Figure 2.png]

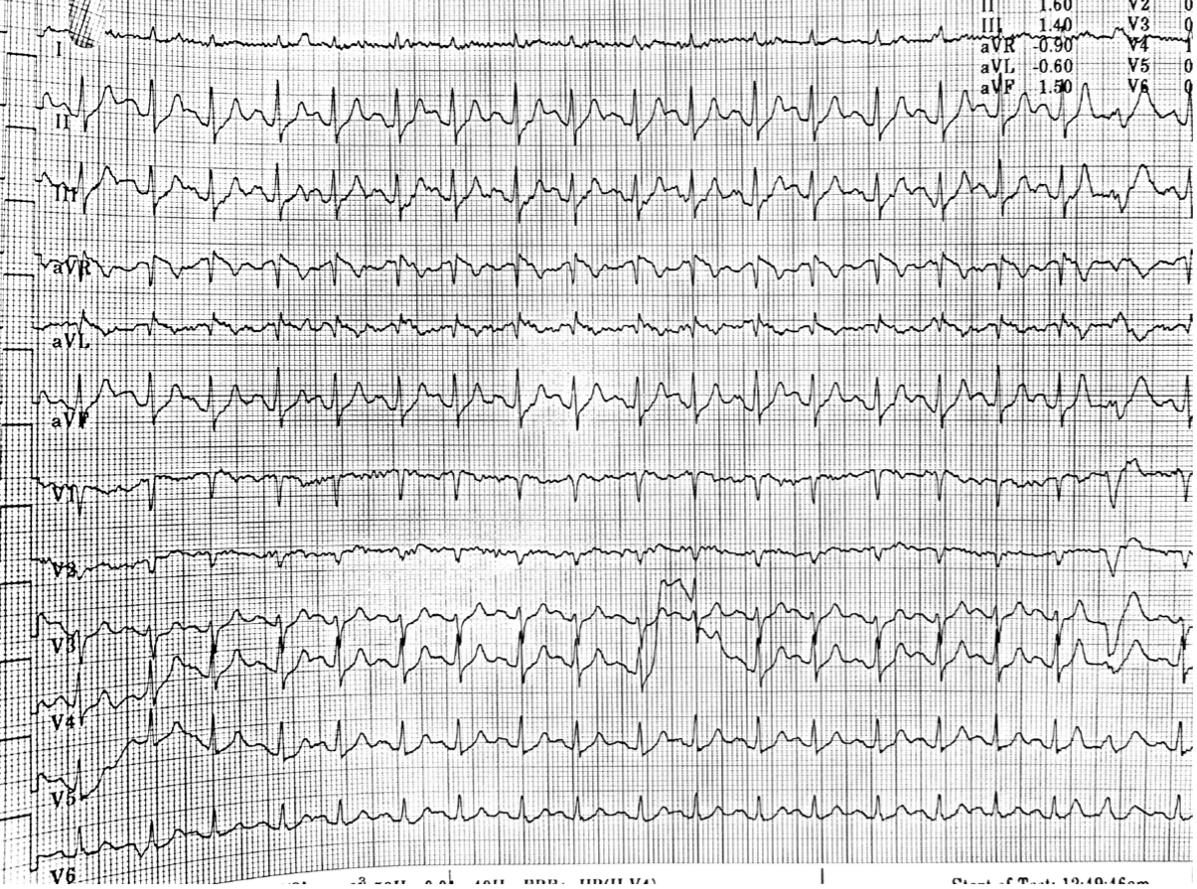

Supplement: ytaf075_Supplementary_Data [file ytaf075_Supplementary_Data.zip › sup figure 4 .png]

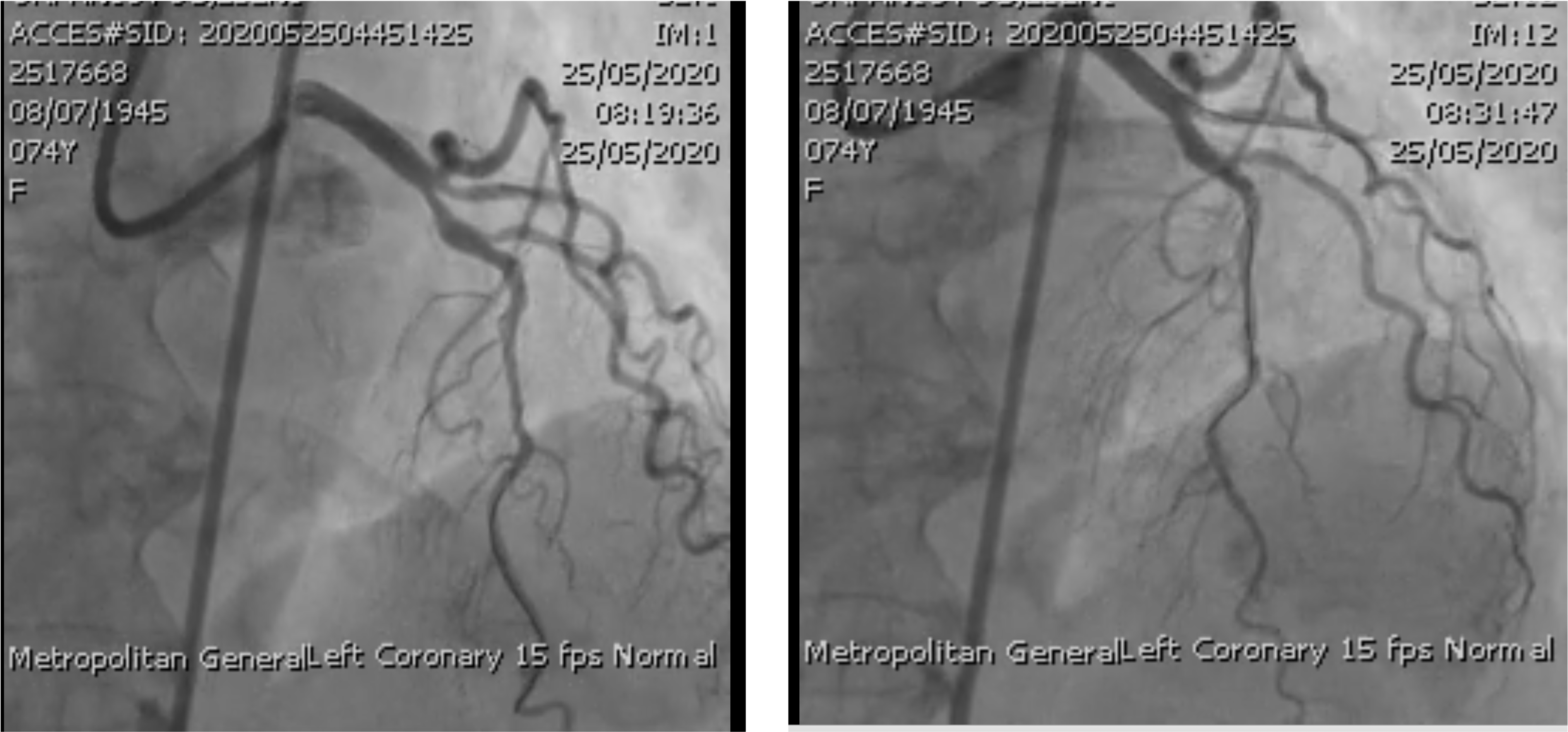

Supplement: ytaf075_Supplementary_Data [file ytaf075_Supplementary_Data.zip › sup Figure 5 revision 2024.png]
